# Supplementary material for: A UMLS-based spell checker for natural language processing in vaccine safety
Source: BMC Med Inform Decis Mak. 2007 Feb 12;7:3. doi: 10.1186/1472-6947-7-3 (PMC1805499; doi:10.1186/1472-6947-7-3)
Supplement: Additional file 13 — RAP application directory. Sets up directory for RAP (RDF application for PHP) source code [file 1472-6947-7-3-S13.gz › rap/doc/phpdoc/__filesource/fsource_syntax__syntaxRdfParser.php.html]

xml version="1.0" encoding="iso-8859-1"?


File Source for RdfParser.php


# Source for file RdfParser.php

Documentation is available at RdfParser.php

```
1. <?php

3. // ----------------------------------------------------------------------------------
4. // Class: RdfParser
5. // ----------------------------------------------------------------------------------


11. /**
12. * An RDF paser.
13. * This class reads RDF data from files or URIs and generates models out of it. All valid
14. * RDF XML syntaxes defined by the W3C in RDF/XML Syntax Specification (Revised)
15. * - W3C Working Draft 10 October 2003
16. * (http://www.w3.org/TR/2003/WD-rdf-syntax-grammar-20031010/) are supported.
17. * The parser is based on the PHP version of repat
18. * (http://phpxmlclasses.sourceforge.net/show_doc.php?class=class_rdf_parser.html)
19. * by Luis Argerich (lrargerich@yahoo.com).
20. *
21. * <BR><BR>History:<UL>
22. * <LI>12-06-2004                : improved namespace handling added (tobias.gauss@web.de)</LI>
23. * <LI>08-13-2004                 : Bug in rdf:resource="" fixed</LI>
24. * <LI>08-10-2004                : function for converting strings to its unicode NFC form added. Benjamin Nowack <bnowack@appmosphere.com></LI>
25. * <LI>06-01-2004                 : Bug in xml:lang fixed, some bugs in the handling of collections fixed</LI>
26. * <LI>05-03-2004                 : generateModel(): added optional parameter $rdfBaseURI to set the rdf base URI manually for strings when there's
27. *                                        no xml:base stated <mail@d-westphal.de>.</LI>
28. * <LI>11-18-2003                 : Made compliant with the latest RDF Specification (10. October 2003) <radol@gmx.de>.
29. *
30. *                                           Support for several aspects of the RDF Specification
31. *                                        was added or fixed, among others:
32. *                                            bugs in handling of xml:base and resolving relative URIs fixed,
33. *                                            support for parseType="Literal" (XMLLiterals) added,
34. *                                            support for parseType="Collection" added,
35. *                                            handling of xml:lang improved,
36. *                                            handling of containers improved,
37. *                                            handling of empty property elements improved,
38. *                                            handling of RDF vocabulary improved,
39. *                                            some cases of reification fixed,
40. *
41. *                                   Several methods have been changed, unused variables and
42. *                                           parameters removed (see CVS for details).
43. *                                   new private methods: _is_forbidden_rdf_property_attribute(),
44. *                                                        _is_forbidden_rdf_property_element ($local_name),
45. *                                                        _is_rdf_node_element(),
46. *                                                        _is_forbidded_node_element(),
47. *                                                        _report_error(),
48. *                                                        _handle_collection_element(),
49. *                                                        _handle_xml_start_element(),
50. *                                                        _handle_xml_end_element(),
51. *                                                        _join_name_and_declare_prefix(),
52. *                                                        _end_collection(),
53. *                                                        _start_ns_declaration_handler(),
54. *                                   removed private methods: _delete_elements(),
55. *                                                            _end_empty_resource_property(),
56. *                                                            rdf_resolve_uri()
57. *
58. *                                   BUGS WHICH HAVE NOT BEED FIXED:
59. *                                           - correct handling of non US-ASCII characters</LI>
60. *
61. * <LI>07-27-2003                : Functions addapted to the new class tree (MemModel extends Model)</LI>
62. * <LI>04-23-2003                : Bug concerning bnode recognition fixed.</LI>
63. * <LI>04-03-2003                : Bug concerning bnode identifier generation fixed.</LI>
64. * <LI>01-10-2003                : Support for rdf:datatype and rdf:nodeID added.</LI>
65. * <LI>01-05-2003                : Support for rdf:seeAlso added.</LI>
66. * <LI>11-07-2002                : Possibility to pass RDF code  directly to the method generateModel() added.</LI>
67. * <LI>10-25-2002                : Bug concerning 1 character long literals fixed.</LI>
68. * <LI>09-24-2002                : Recognition of blank nodes improved.</LI>
69. * <LI>09-10-2002                : First version of this class.</LI>
70. * </UL>
71. * @version  V0.9.1
72. * @author Luis Argerich <lrargerich@yahoo.com>,
73. *          Chris Bizer <chris@bizer.de>,
74. *          Radoslaw Oldakowski <radol@gmx.de>
75. *            Daniel Westphal <mail@d-westphal.de>
76. * @package syntax
77. * @access    public
78. *
79. */

81. class RdfParser extends Object {

83. var $rdf_parser;
84. var $model;

86. /* Private Methods */


89. /**
90. * converts a string to its unicode NFC form (e.g. \uHHHH or \UHHHHHHHH).
91. *
92. * @param String $str
93. * @return String
94. * @access private
95. *
96. */
97. function str2unicode_nfc($str=""){
98. $result="";
99. /* try to detect encoding */
100. $tmp=str_replace("?", "", $str);
101. if(strpos(utf8_decode($tmp), "?")===false){
102. $str=utf8_decode($str);
103. }
104. for($i=0,$i_max=strlen($str);$i<$i_max;$i++){
105. $nr=0;/* unicode dec nr */
106. /* char */
107. $char=$str[$i];
108. /* utf8 binary */
109. $utf8_char=utf8_encode($char);
110. $bytes=strlen($utf8_char);
111. if($bytes==1){
112. /* 0####### (0-127) */
113. $nr=ord($utf8_char);
114. }
115. elseif($bytes==2){
116. /* 110##### 10###### = 192+x 128+x */
117. $nr=((ord($utf8_char[0])-192)*64) + (ord($utf8_char[1])-128);
118. }
119. elseif($bytes==3){
120. /* 1110#### 10###### 10###### = 224+x 128+x 128+x */
121. $nr=((ord($utf8_char[0])-224)*4096) + ((ord($utf8_char[1])-128)*64) + (ord($utf8_char[2])-128);
122. }
123. elseif($bytes==4){
124. /* 1111#### 10###### 10###### 10###### = 240+x 128+x 128+x 128+x */
125. $nr=((ord($utf8_char[0])-240)*262144) + ((ord($utf8_char[1])-128)*4096) + ((ord($utf8_char[2])-128)*64) + (ord($utf8_char[3])-128);
126. }
127. /* result (see http://www.w3.org/TR/rdf-testcases/#ntrip_strings) */
128. if($nr<9){/* #x0-#x8 (0-8) */
129. $result.="\\u".sprintf("%04X",$nr);
130. }
131. elseif($nr==9){/* #x9 (9) */
132. $result.='\t';
133. }
134. elseif($nr==10){/* #xA (10) */
135. $result.='\n';
136. }
137. elseif($nr<13){/* #xB-#xC (11-12) */
138. $result.="\\u".sprintf("%04X",$nr);
139. }
140. elseif($nr==13){/* #xD (13) */
141. $result.='\t';
142. }
143. elseif($nr<32){/* #xE-#x1F (14-31) */
144. $result.="\\u".sprintf("%04X",$nr);
145. }
146. elseif($nr<34){/* #x20-#x21 (32-33) */
147. $result.=$char;
148. }
149. elseif($nr==34){/* #x22 (34) */
150. $result.='\"';
151. }
152. elseif($nr<92){/* #x23-#x5B (35-91) */
153. $result.=$char;
154. }
155. elseif($nr==92){/* #x5C (92) */
156. $result.='\\';
157. }
158. elseif($nr<127){/* #x5D-#x7E (93-126) */
159. $result.=$char;
160. }
161. elseif($nr<65536){/* #x7F-#xFFFF (128-65535) */
162. $result.="\\u".sprintf("%04X",$nr);
163. }
164. elseif($nr<1114112){/* #x10000-#x10FFFF (65536-1114111) */
165. $result.="\\U".sprintf("%08X",$nr);
166. }
167. else{
168. /* other chars are not defined => ignore */
169. }
170. }
171. return $result;
172. }


177. /**
178. * @access    private
179. */
180. function _new_element()
181. {
182. $e['parent']=Array();  // Parent is a blank Array
183. $e['state']=0;
184. $e['has_property_atributes']=0;
185. $e['has_member_attributes']=0;
186. $e['subject_type']=0;
187. $e['subject']='';
188. $e['predicate']='';
189. $e['ordinal']=0;
190. $e['members']=0;
191. $e['data']='';
192. $e['xml_lang']='';
193. $e['bag_id']='';
194. $e['statements']=0;
195. $e['statement_id']='';
196. $e['datatype']='';
197. $e['element_base_uri'] = '';

199. return $e;
200. }

202. /**
203. * @param string $source
204. * @param string &$destination
205. *
206. * @access    private
207. */
208. function _copy_element($source, &$destination )
209. {
210. if( $source )
211. {
212. $destination['parent'] = $source;
213. $destination['state'] = $source['state'];
214. $destination['xml_lang'] = $source['xml_lang'];
215. $destination['element_base_uri'] = $source['element_base_uri'];
216. }
217. }

219. /**
220. * @param string &$e
221. * @access    private
222. */
223. function _clear_element(&$e)
224. {
225. $e['subject']='';
226. $e['predicate']='';
227. $e['data']='';
228. $e['bag_id']='';
229. $e['statement_id']='';

231. if(isset($e['parent'])) {
232. if( $e['parent'] )
233. {
234. if( $e['parent']['xml_lang'] != $e['xml_lang'] )
235. {
236. $e['xml_lang']='';
237. }
238. }
239. else
240. {
241. $e['xml_lang']='';
242. }
243. } else {
244. $e['xml_lang']='';
245. }

247. $e['parent']=Array();
248. $e['state']=0;
249. $e['has_property_attributes']=0;
250. $e['has_member_attributes']=0;
251. $e['subject_type']=0;
252. $e['subject']='';
253. $e['predicate']='';
254. $e['ordinal']=0;
255. $e['members']=0;
256. $e['data']='';
257. $e['xml_lang']='';
258. $e['bag_id']='';
259. $e['statements']=0;
260. $e['statement_id']='';
261. $e['datatype']='';
262. $e['element_base_uri'] = '';
263. }

265. /**
266. * @access    private
267. */
268. function _push_element()
269. {
270. if(!isset($this->rdf_parser['free'])) {
271. $this->rdf_parser['free']=Array();
272. }
273. if(count($this->rdf_parser['free'])>0)
274. {
275. $e = $this->rdf_parser['free'];
276. if(isset($e['parent'])) {
277. $this->rdf_parser['free'] = $e['parent'];
278. } else {
279. $this->rdf_parser['free']=$this->_new_element();
280. }
281. }
282. else
283. {
284. $e = $this->_new_element();
285. }
286. if(!isset($this->rdf_parser['top'])) {
287. $this->rdf_parser['top']=Array();
288. }
289. $this->_copy_element( $this->rdf_parser['top'], $e );
290. $this->rdf_parser['top'] = $e;
291. }

293. /**
294. * @access    private
295. */
296. function _pop_element()
297. {
298. $e = $this->rdf_parser['top'];
299. $this->rdf_parser['top'] = $e['parent'];
300. $this->_clear_element( $e );
301. $this->rdf_parser['free'] = $e;
302. }

304. /**
305. * @param string $local_name
306. * @access    private
307. */
308. function _is_rdf_property_attribute_resource($local_name )
309. {
310. return ( $local_name == RDF_TYPE );
311. }

313. /**
314. * @param string $local_name
315. * @access    private
316. */
317. function _is_rdf_property_attribute_literal($local_name )
318. {
319. return ( $local_name == RDF_VALUE )
320. || ( $local_name == RDF_BAG )
321. || ( $local_name == RDF_SEQ )
322. || ( $local_name == RDF_ALT )
323. || ( $local_name == RDF_STATEMENT )
324. || ( $local_name == RDF_PROPERTY )
325. || ( $local_name == RDF_LIST );
326. }

328. /**
329. * @param string $local_name
330. * @access    private
331. */
332. function _is_rdf_ordinal( $local_name )
333. {
334. $ordinal = -1;

336. if( $local_name{0} ==  '_'  )
337. {
338. $ordinal =  substr($local_name,1) + 1 ;
339. }

341. return ( $ordinal > 0 ) ? $ordinal : 0;
342. }

344. /**
345. * @param string $local_name
346. * @access    private
347. */
348. function _is_rdf_property_attribute( $local_name )
349. {
350. return $this->_is_rdf_property_attribute_resource( $local_name )
351. || $this->_is_rdf_property_attribute_literal( $local_name );
352. }

354. function _is_forbidden_rdf_property_attribute($local_name)
355. {
356. return ( $local_name == RDF_RDF )
357. || ( $local_name == RDF_DESCRIPTION)
358. || ( $local_name == RDF_ID)
359. || ( $local_name == RDF_ABOUT )
360. || ( $local_name == RDF_BAG_ID )
361. || ( $local_name == RDF_PARSE_TYPE )
362. || ( $local_name == RDF_RESOURCE )
363. || ( $local_name == RDF_NODEID )
364. || ( $local_name == RDF_LI )
365. || ( $local_name == RDF_ABOUT_EACH )
366. || ( $local_name == RDF_ABOUT_EACH_PREFIX )
367. || ( $local_name == RDF_DATATYPE );
368. }

370. /**
371. * @param string $local_name
372. * @access    private
373. */
374. function _is_rdf_property_element( $local_name )
375. {
376. return (  $local_name == RDF_TYPE )
377. || (  $local_name == RDF_SUBJECT )
378. || (  $local_name == RDF_PREDICATE )
379. || (  $local_name == RDF_OBJECT )
380. || (  $local_name == RDF_VALUE )
381. || (  $local_name == RDF_LI )
382. || (  $local_name == RDF_SEEALSO )
383. || ( $local_name == RDF_BAG )
384. || ( $local_name == RDF_SEQ )
385. || ( $local_name == RDF_ALT )
386. || ( $local_name == RDF_STATEMENT )
387. || ( $local_name == RDF_PROPERTY )
388. || ( $local_name == RDF_LIST )
389. || ( $local_name == RDF_FIRST )
390. || ( $local_name == RDF_REST )
391. || (  $local_name{0} == '_'  );
392. }

394. /**
395. * @param string $local_name
396. * @access    private
397. */
398. function _is_forbidden_rdf_property_element ($local_name)
399. {
400. return ( $local_name == RDF_RDF )
401. || ( $local_name == RDF_DESCRIPTION)
402. || ( $local_name == RDF_ID)
403. || ( $local_name == RDF_ABOUT )
404. || ( $local_name == RDF_BAG_ID )
405. || ( $local_name == RDF_PARSE_TYPE )
406. || ( $local_name == RDF_RESOURCE )
407. || ( $local_name == RDF_NODEID )
408. || ( $local_name == RDF_ABOUT_EACH )
409. || ( $local_name == RDF_ABOUT_EACH_PREFIX )
410. || ( $local_name == RDF_DATATYPE );
411. }


414. /**
415. * @param string $local_name
416. * @access    private
417. */
418. function _is_rdf_node_element( $local_name )
419. {
420. return ( $local_name == RDF_DESCRIPTION )
421. || ( $local_name == RDF_STATEMENT )
422. || ( $local_name == RDF_SUBJECT )
423. || ( $local_name == RDF_PREDICATE )
424. || ( $local_name == RDF_OBJECT )
425. || ( $local_name == RDF_PROPERTY )
426. || ( $local_name == RDF_TYPE )
427. || ( $local_name == RDF_VALUE )
428. || ( $local_name == RDF_BAG )
429. || ( $local_name == RDF_SEQ )
430. || ( $local_name == RDF_ALT )
431. || ( $local_name == RDF_SEEALSO )
432. || ( $local_name == RDF_LIST )
433. || ( $local_name == RDF_FIRST )
434. || ( $local_name == RDF_REST )
435. || ( $local_name == RDF_NIL )
436. || ( $local_name{0} == '_'  );
437. }

439. /**
440. * @param string $local_name
441. * @access    private
442. */
443. function _is_forbidden_rdf_node_element ($local_name)
444. {
445. return ( $local_name == RDF_RDF )
446. || ( $local_name == RDF_ID)
447. || ( $local_name == RDF_ABOUT )
448. || ( $local_name == RDF_BAG_ID )
449. || ( $local_name == RDF_PARSE_TYPE )
450. || ( $local_name == RDF_RESOURCE )
451. || ( $local_name == RDF_NODEID )
452. || ( $local_name == RDF_LI )
453. || ( $local_name == RDF_ABOUT_EACH )
454. || ( $local_name == RDF_ABOUT_EACH_PREFIX )
455. || ( $local_name == RDF_DATATYPE );
456. }

458. /**
459. * @param string $val
460. * @access    private
461. */
462. function _istalnum($val) {
463. return ereg("[A-Za-z0-9]",$val);
464. }
465. /**
466. * @param string $val
467. * @access    private
468. */
469. function _istalpha($val) {
470. return ereg("[A-Za-z]",$val);
471. }

473. /**
474. * @param string $uri
475. * @access    private
476. */
477. function _is_absolute_uri($uri )
478. {
479. $result = false;
480. $uri_p=0;
481. if( $uri && $this->_istalpha( $uri{$uri_p} ) )
482. {
483. ++$uri_p;

485. while( ($uri_p<strlen($uri))
486. && ( $this->_istalnum( $uri{$uri_p} )
487. || ( $uri{$uri_p} ==  '+'  )
488. || ( $uri{$uri_p} == '-'  )
489. || ( $uri{$uri_p} ==  '.'  ) ) )
490. {
491. ++$uri_p;
492. }

494. $result = ( $uri{$uri_p} == ':'  );
495. }
496. return $result;
497. }


500. /*
501. * This function returns an associative array returning any of the various components of the URL that are present. This includes the
502. * $arr=parse_url($url)
503. * scheme - e.g. http
504. * host
505. * port
506. * user
507. * pass
508. * path
509. * query - after the question mark ?
510. * fragment - after the hashmark #
511. *
512. * @param string $uri
513. * @param string $buffer
514. * @param string &$scheme
515. * @param string &$authority
516. * @param string &$path
517. * @param string &$query
518. * @param string &$fragment
519. * @access    private
520. */
521. function _parse_uri($uri,$buffer,&$scheme,&$authority,&$path,&$query,&$fragment ) {

523. $parsed=parse_url($uri);
524. if(isset($parsed['scheme'])) {
525. $scheme=$parsed['scheme'];
526. } else {
527. $scheme='';
528. }
529. if(isset($parsed['host'])) {
530. $host=$parsed['host'];
531. } else {
532. $host='';
533. }
534. if(isset($parsed['host'])) {
535. $authority=$parsed['host'];
536. } else {
537. $authority='';
538. }
539. if(isset($parsed['path'])) {
540. $path=$parsed['path'];
541. } else {
542. $path='';
543. }
544. if(isset($parsed['query'])) {
545. $query=$parsed['query'];
546. } else {
547. $query='';
548. }
549. if(isset($parsed['fragment'])) {
550. $fragment=$parsed['fragment'];
551. } else {
552. $fragment='';
553. }

555. }

557. /**
558. * @param string $base_uri
559. * @param string $reference_uri
560. * @param string &$buffer
561. * @access    private
562. */
563. function _resolve_uri_reference($base_uri,$reference_uri,&$buffer )
564. {
565. if ($reference_uri == '')
566. return ($buffer = preg_replace("/\#[^\/\\\]*$/", '', $base_uri));

568. $base_buffer='';
569. $reference_buffer='';
570. $path_buffer='';

572. $buffer = '';

574. $this->_parse_uri($reference_uri,
575. $reference_buffer,
576. $reference_scheme,
577. $reference_authority,
578. $reference_path,
579. $reference_query,
580. $reference_fragment );

582. $this->_parse_uri($base_uri,
583. $base_buffer,
584. $base_scheme,
585. $base_authority,
586. $base_path,
587. $base_query,
588. $base_fragment );

590. if( $reference_scheme == ''
591. && $reference_authority == ''
592. && $reference_path == ''
593. && $reference_query == '' )
594. {
595. $buffer=$base_uri;

597. if( $reference_fragment != '' )
598. {
599. if ($base_path == '' || $base_path == '/' || $base_path == "\\") {
600. $buffer = $this->rdf_parser['document_base_uri'];
601. }
602. else
603. {
604. $buffer = preg_replace("/\#[^\/\\\]*$/", '', $base_uri);
605. }

607. // CB: Changed for base URI
608. $c = substr($buffer, strlen($buffer)-1 ,1);
609. if (!($c=='#' || $c==':' || $c=='/' || $c=="\\"))
610. $buffer.= '#' ;
611. $buffer.=$reference_fragment;

613. }
614. }
615. else if( $reference_scheme != '' )
616. {
617. $buffer=$reference_uri;
618. }
619. else
620. {
621. $result_scheme = $base_scheme;
622. $result_path = '';

624. if( $reference_authority != '' )
625. {
626. $result_authority = $reference_authority;
627. }
628. else
629. {
630. $result_authority = $base_authority;

632. if ($reference_path != '')
633. {
634. if ($reference_path{0} == '/' || $reference_path{0} == "\\")
635. {
636. if ($reference_path{1} == '/' || $reference_path{1} == "\\")
637. {
638. $result_authority = '';
639. $result_path = $reference_path;
640. }
641. else
642. $result_path = $reference_path;
643. }
644. elseif (substr($reference_path, 0, 3) == '../' ||
645. substr($reference_path, 0, 3) == '..\\')
646. {
647. $slash = $reference_path{2};
648. while($base_path != '' && ( substr($reference_path, 0, 3) == '../'
649. || substr($reference_path, 0, 3) == '..\\'))
650. {
651. $base_path = preg_replace("/((\/)|(\\\))[^\/\\\]*$/", '', $base_path);
652. if ($base_path != '') {
653. $base_path = preg_replace("/((\/)|(\\\))[^\/\\\]*$/", '', $base_path);
654. $reference_path = substr($reference_path, 3);
655. }
656. }

658. $result_path = $base_path .$slash .$reference_path;
659. }
660. else
661. {
662. if ($base_path)
663. $result_path = preg_replace("/[^\/\\\]*$/", $reference_path, $base_path, 1);
664. else
665. $result_path = '/' .$reference_path;
666. }
667. }

669. }

671. if( $result_scheme != '' )
672. {
673. $buffer=$result_scheme;
674. $buffer.=':';
675. }

677. if( $result_authority != '' )
678. {
679. $buffer.="//";
680. $buffer.=$result_authority;
681. }

683. if( $result_path != '' )
684. {
685. $buffer.=$result_path;
686. }

688. if( $reference_query != '' )
689. {
690. $buffer.='?';
691. $buffer.=$reference_query;
692. }

694. if( $reference_fragment != '' )
695. {
696. $buffer.='#';
697. $buffer.=$reference_fragment;
698. }
699. }
700. }


703. /**
704. * IDs which contain CombiningChars or Extenders
705. * (see http://www.w3.org/TR/REC-xml-names/#NT-NCName) are assumed to be invalid.
706. * If you want to use IDs containing these characters you can turn off
707. * the validating by setting the constant VALIDATE_IDS to FALSE (see constants.php).
708. *
709. * @param string $id
710. * @access    private
711. */
712. function is_valid_id($id )
713. {
714. if (!VALIDATE_IDS)
715. return TRUE;

717. $result = FALSE;

719. if( $id )
720. {
721. if( $this->_istalpha($id{0}) || $id{0} == '_')
722. {
723. $result = TRUE;
724. $i=0;
725. $len = strlen($id);
726. while( $result != FALSE && ++$i < $len )
727. {
728. if( !($this->_istalnum( $id{$i})
729. || $id{$i} == '.'
730. || $id{$i} == '-'
731. || $id{$i} == '_'))
732. {
733. $result = FALSE;
734. }
735. }
736. }
737. }

739. if (!$result)
740. $this->_report_error('illegal ID, nodeID or bagID attribute value');
741. else
742. return TRUE;
743. }

745. /**
746. * @param string $id
747. * @param string &$buffer
748. * @access    private
749. */
750. function _resolve_id($id,&$buffer )
751. {
752. $id_buffer='';

754. if( $this->is_valid_id($id) )
755. {
756. $id_buffer="#$id";
757. }

759. $this->_resolve_uri_reference( $this->rdf_get_base(), $id_buffer, $buffer );

761. }

763. /**
764. * @param string $name
765. * @param string &$buffer
766. * @param string &$namespace_uri
767. * @param string &$local_name
768. * @access    private
769. */
770. function _split_name($name, &$buffer, &$namespace_uri, &$local_name )
771. {
772. static $nul = 0;
773. $buffer=$name;

775. if(  strstr( $buffer, NAMESPACE_SEPARATOR_CHAR ) )
776. {
777. $cosas=explode(NAMESPACE_SEPARATOR_CHAR,$buffer);
778. $namespace_uri = $cosas[0];
779. $local_name = $cosas[1];
780. }
781. else
782. {
783. if( ( $buffer{ 0 } ==  'x'  )
784. && ( $buffer{ 1 } ==  'm'  )
785. && ( $buffer{ 2 } ==  'l'  )
786. && ( $buffer{ 3 } ==  ':'  ) )
787. {
788. $namespace_uri = XML_NAMESPACE_URI;
789. $local_name = substr($buffer,4);
790. }
791. else
792. {
793. $namespace_uri = '';
794. $local_name = $buffer;
795. }
796. }
797. }
798. /**
799. * @param string &$buf
800. * @access    private
801. */
802. function _generate_anonymous_uri(&$buf )
803. {
804. $id='';
805. if(!isset($this->rdf_parser['anonymous_id'])) {
806. $this->rdf_parser['anonymous_id']=0;
807. }
808. $this->rdf_parser['anonymous_id']++;

810. $buf= BNODE_PREFIX . $this->rdf_parser['anonymous_id'];

812. }
813. /**
814. * @param string $subject_type
815. * @param string $subject
816. * @param string $predicate
817. * @param string $ordinal
818. * @param string $object_type
819. * @param string $object
820. * @param string $xml_lang
821. * @param string $bag_id
822. * @param string $statements
823. * @param string $statement_id
824. * @access    private
825. */
826. function _report_statement( $subject_type, $subject, $predicate, $ordinal, $object_type,  $object, $xml_lang, $bag_id, $statements, $statement_id, $datatype )
827. {
828. $statement_id_type = RDF_SUBJECT_TYPE_URI;
829. $statement_id_buffer='';
830. $predicate_buffer='';
831. if (!$xml_lang && $object_type == RDF_OBJECT_TYPE_LITERAL && isset($this->rdf_parser['document_xml_lang']))
832. $xml_lang = $this->rdf_parser['document_xml_lang'];

834. // call add statement
835. $this->add_statement_to_model($this->rdf_parser['user_data'],$subject_type,$subject,$predicate,$ordinal,$object_type,$object,$xml_lang, $datatype );

837. if( $bag_id )
838. {
839. if( $statements == '' )
840. {
841. $this->_report_statement(RDF_SUBJECT_TYPE_URI,
842. $bag_id,
843. RDF_NAMESPACE_URI.RDF_TYPE,
844. 0,
845. RDF_OBJECT_TYPE_RESOURCE,
846. RDF_NAMESPACE_URI.RDF_BAG,
847. '',
848. '',
849. '',
850. '',
851. $datatype );
852. }

854. if( ! $statement_id )
855. {
856. $statement_id_type = RDF_SUBJECT_TYPE_BNODE;
857. $this->_generate_anonymous_uri( $statement_id_buffer );
858. $statement_id = $statement_id_buffer;
859. }
860. $statements++;
861. $predicate_buffer='RDF_NAMESPACE_URI_'.$statements;

863. $this->_report_statement(
864. RDF_SUBJECT_TYPE_URI,
865. $bag_id,
866. $predicate_buffer,
867. $statements,
868. RDF_OBJECT_TYPE_BNODE,
869. $statement_id,
870. '',
871. '',
872. '',
873. '',
874. $datatype );
875. }

877. if( $statement_id )
878. {
879. // rdf:type = rdf:Statement
880. $this->_report_statement(
881. $statement_id_type,
882. $statement_id,
883. RDF_NAMESPACE_URI.RDF_TYPE,
884. 0,
885. RDF_OBJECT_TYPE_RESOURCE,
886. RDF_NAMESPACE_URI.RDF_STATEMENT,
887. '',
888. '',
889. '',
890. '',
891. $datatype );

893. if ($subject_type == RDF_SUBJECT_TYPE_BNODE)
894. $obj_type = RDF_OBJECT_TYPE_BNODE;
895. else
896. $obj_type = RDF_OBJECT_TYPE_RESOURCE;


899. // rdf:subject
900. $this->_report_statement(
901. $statement_id_type,
902. $statement_id,
903. RDF_NAMESPACE_URI.RDF_SUBJECT,
904. 0,
905. $obj_type,
906. $subject,
907. '',
908. '',
909. '',
910. '',
911. $datatype );

913. // rdf:predicate
914. $this->_report_statement(
915. $statement_id_type,
916. $statement_id,
917. RDF_NAMESPACE_URI.RDF_PREDICATE,
918. 0,
919. RDF_OBJECT_TYPE_RESOURCE,
920. $predicate,
921. '',
922. '',
923. '',
924. '',
925. $datatype );

927. // rdf:object
928. $this->_report_statement(
929. $statement_id_type,
930. $statement_id,
931. RDF_NAMESPACE_URI.RDF_OBJECT,
932. 0,
933. $object_type,
934. $object,
935. '',
936. '',
937. '',
938. '',
939. $datatype );
940. }
941. }
942. /**
943. * @param string $subject_type
944. * @param string $subject
945. * @param string $attributes
946. * @param string $xml_lang
947. * @param string $bag_id
948. * @param string $statements
949. * @access    private
950. */
951. function _handle_property_attributes($subject_type, $subject, $attributes, $xml_lang, $bag_id, $statements )
952. {
953. $i=0;

955. $attribute='';
956. $predicate='';

958. $attribute_namespace_uri='';
959. $attribute_local_name='';
960. $attribute_value='';

962. $ordinal=0;

964. for( $i = 0; isset($attributes[ $i ]); $i += 2 )
965. {
966. $this->_split_name(
967. $attributes[ $i ],
968. $attribute,
969. $attribute_namespace_uri,
970. $attribute_local_name );

972. $attribute_value = $attributes[ $i + 1 ];

974. $predicate=$attribute_namespace_uri;
975. $predicate.=$attribute_local_name;

977. if( RDF_NAMESPACE_URI == $attribute_namespace_uri )
978. {
979. if( $this->_is_rdf_property_attribute_literal( $attribute_local_name ) )
980. {
981. $this->_report_statement(
982. $subject_type,
983. $subject,
984. $predicate,
985. 0,
986. RDF_OBJECT_TYPE_LITERAL,
987. $attribute_value,
988. $xml_lang,
989. $bag_id,
990. $statements,
991. '',
992. '');
993. }
994. else if( $this->_is_rdf_property_attribute_resource( $attribute_local_name ) )
995. {
996. $this->_report_statement(
997. $subject_type,
998. $subject,
999. $predicate,
1000. 0,
1001. RDF_OBJECT_TYPE_RESOURCE,
1002. $attribute_value,
1003. '',
1004. $bag_id,
1005. $statements,
1006. '',
1007. '');
1008. }
1009. else if( ( $ordinal = $this->_is_rdf_ordinal( $attribute_local_name ) ) != 0 )
1010. {
1011. $this->_report_statement(
1012. $subject_type,
1013. $subject,
1014. $predicate,
1015. $ordinal,
1016. RDF_OBJECT_TYPE_LITERAL,
1017. $attribute_value,
1018. $xml_lang,
1019. $bag_id,
1020. $statements,
1021. '',
1022. '' );
1023. }
1024. else if (   ($attribute_local_name != RDF_ABOUT)
1025. && ($attribute_local_name != RDF_RDF)
1026. && ($attribute_local_name != RDF_DESCRIPTION)
1027. && ($attribute_local_name != RDF_ID)
1028. && ($attribute_local_name != RDF_ABOUT_EACH)
1029. && ($attribute_local_name != RDF_ABOUT_EACH_PREFIX)
1030. && ($attribute_local_name != RDF_BAG_ID)
1031. && ($attribute_local_name != RDF_RESOURCE)
1032. && ($attribute_local_name != RDF_PARSE_TYPE)
1033. && ($attribute_local_name != RDF_PARSE_TYPE_LITERAL)
1034. && ($attribute_local_name != RDF_PARSE_TYPE_RESOURCE)
1035. && ($attribute_local_name != RDF_LI)
1036. && ($attribute_local_name != RDF_SUBJECT)
1037. && ($attribute_local_name != RDF_PREDICATE)
1038. && ($attribute_local_name != RDF_OBJECT)
1039. && ($attribute_local_name != RDF_NODEID)
1040. && ($attribute_local_name != RDF_DATATYPE)
1041. && ($attribute_local_name != RDF_SEEALSO)
1042. && ($attribute_local_name != RDF_NIL)
1043. && ($attribute_local_name != RDF_REST)
1044. && ($attribute_local_name != RDF_FIRST)
1045. )
1046. {
1047. $this->_report_statement(
1048. $subject_type,
1049. $subject,
1050. $predicate,
1051. 0,
1052. RDF_OBJECT_TYPE_LITERAL,
1053. $attribute_value,
1054. $xml_lang,
1055. $bag_id,
1056. $statements,
1057. '',
1058. '' );
1059. }
1060. }
1061. else if( XML_NAMESPACE_URI == $attribute_namespace_uri )
1062. {
1063. if ($attribute_local_name == 'base')
1064. {
1065. $this->rdf_parser['top']['element_base_uri'] = $attribute_value;
1066. }
1067. }
1068. else if( $attribute_namespace_uri )
1069. {
1070. // is it required that property attributes be in an explicit namespace?

1072. $this->_report_statement(
1073. $subject_type,
1074. $subject,
1075. $predicate,
1076. 0,
1077. RDF_OBJECT_TYPE_LITERAL,
1078. $attribute_value,
1079. $xml_lang,
1080. $bag_id,
1081. $statements,
1082. '',
1083. '' );
1084. }
1085. }
1086. }


1090. /**
1091. * @param string $warning
1092. * @access    private
1093. */
1094. function _report_warning($warning)
1095. {
1096. $errmsg = RDFAPI_ERROR . '(class: parser): ' . $warning .'.';
1097. trigger_error($errmsg, E_USER_WARNING);
1098. }


1101. function _report_error($error)
1102. {
1103. $errmsg = RDFAPI_ERROR . '(class: parser): ' . $error .'.';
1104. trigger_error($errmsg, E_USER_ERROR);
1105. }


1108. /**
1109. * @param string $namespace_uri
1110. * @param string $local_name
1111. * @param string $attributes
1112. * @param string $parent
1113. * @access    private
1114. */
1115. function _handle_resource_element( $namespace_uri, $local_name, $attributes, $parent )
1116. {
1117. $subjects_found = 0;
1118. $aux=$attributes;
1119. $aux2=Array();
1120. foreach($attributes as $atkey=>$atvalue) {
1121. $aux2[]=$atkey;
1122. $aux2[]=$atvalue;
1123. }
1124. $attributes=$aux2;
1125. $id = '';
1126. $about = '';

1128. $bag_id = '';
1129. $node_id = '';
1130. $datatype = '';

1132. $i=0;

1134. $attribute='';

1136. $attribute_namespace_uri='';
1137. $attribute_local_name='';
1138. $attribute_value='';

1140. $id_buffer='';

1142. $type='';

1144. $this->rdf_parser['top']['has_property_attributes'] = false;
1145. $this->rdf_parser['top']['has_member_attributes'] = false;


1148. if( $namespace_uri == RDF_NAMESPACE_URI )
1149. {
1150. if( ! $this->_is_rdf_node_element( $local_name ) )
1151. {
1152. $msg = 'unknown or out of context rdf node element: '.$local_name;

1154. if ($this->_is_forbidden_rdf_node_element($local_name))
1155. $this->_report_error($msg);
1156. else
1157. $this->_report_warning($msg);
1158. }
1159. }

1161. // examine each attribute for the standard RDF "keywords"
1162. for( $i = 0; isset($attributes[$i]); $i += 2 )
1163. {
1164. $this->_split_name(
1165. $attributes[ $i ],
1166. $attribute,
1167. $attribute_namespace_uri,
1168. $attribute_local_name );

1170. $attribute_value = $attributes[ $i + 1 ];

1172. // if the attribute is not in any namespace
1173. //   or the attribute is in the RDF namespace
1174. if( ( $attribute_namespace_uri == '' )
1175. || (  $attribute_namespace_uri == RDF_NAMESPACE_URI  ))
1176. {
1177. if( $attribute_local_name == RDF_ID )
1178. {
1179. $id = $attribute_value;
1180. ++$subjects_found;
1181. } else if( $attribute_local_name == RDF_ABOUT ) {
1182. $about = '_'.$attribute_value;
1183. ++$subjects_found;
1184. } else if( $attribute_local_name == RDF_NODEID) {
1185. $node_id = $attribute_value;
1186. ++$subjects_found;
1187. } else if(  $attribute_local_name == RDF_ABOUT_EACH ) {
1188. $error = 'aboutEach has been removed from the RDF specifications';
1189. $this->_report_error($error);
1190. } else if( $attribute_local_name == RDF_ABOUT_EACH_PREFIX ) {
1191. $error = 'aboutEachPrefix has been removed from the RDF specifications';
1192. $this->_report_error($error);
1193. } else if( $attribute_local_name == RDF_BAG_ID) {
1194. $bag_id = $attribute_value;
1195. } else if( $attribute_local_name == RDF_DATATYPE) {
1196. $datatype = $attribute_value;
1197. } else if( $this->_is_rdf_property_attribute( $attribute_local_name )) {
1198. $this->rdf_parser['top']['has_property_attributes'] = true;
1199. } else if( $this->_is_rdf_ordinal( $attribute_local_name )) {
1200. $this->rdf_parser['top']['has_property_attributes'] = true;
1201. $this->rdf_parser['top']['has_member_attributes'] = true;
1202. } else {
1203. $this->rdf_parser['top']['has_property_attributes'] = true;
1204. $msg = 'unknown or out of context rdf attribute: '.$attribute_local_name;

1206. if ($this->_is_forbidden_rdf_property_attribute($attribute_local_name))
1207. $this->_report_error($msg);
1208. else
1209. $this->_report_warning($msg);
1210. }
1211. }
1212. else if(  $attribute_namespace_uri == XML_NAMESPACE_URI )
1213. {
1214. if( $attribute_local_name == XML_LANG )
1215. {
1216. $this->rdf_parser['top']['xml_lang'] = $attribute_value;
1217. }
1218. elseif ($attribute_local_name == 'base')
1219. {
1220. $this->rdf_parser['top']['element_base_uri'] = $attribute_value;
1221. }
1222. }
1223. else if( $attribute_namespace_uri )
1224. {
1225. $this->rdf_parser['top']['has_property_attributes'] = true;
1226. }
1227. }

1229. // if no subjects were found, generate one.
1230. if( $subjects_found == 0 )
1231. {
1232. $this->_generate_anonymous_uri( $id_buffer );
1233. $this->rdf_parser['top']['subject']=$id_buffer;
1234. $this->rdf_parser['top']['subject_type'] = RDF_SUBJECT_TYPE_BNODE;
1235. }
1236. else if( $subjects_found > 1 )
1237. {
1238. $this->_report_error('ID, about and nodeID are mutually exclusive');
1239. }
1240. else if( $id )
1241. {
1242. $this->_resolve_id( $id, $id_buffer );
1243. $this->rdf_parser['top']['subject_type'] = RDF_SUBJECT_TYPE_URI;
1244. $this->rdf_parser['top']['subject']=$id_buffer;
1245. }
1246. else if( $about )
1247. {
1248. $this->_resolve_uri_reference( $this->rdf_get_base(), substr($about,1), $id_buffer );
1249. $this->rdf_parser['top']['subject_type'] = RDF_SUBJECT_TYPE_URI;
1250. $this->rdf_parser['top']['subject']=$id_buffer;
1251. }
1252. else if( $node_id )
1253. {
1254. $this->is_valid_id($node_id);
1255. $this->rdf_parser['top']['subject_type'] = RDF_SUBJECT_TYPE_BNODE;
1256. $this->rdf_parser['top']['subject']=$node_id;
1257. }

1259. // if the subject is empty, assign it the document uri
1260. if( $this->rdf_parser['top']['subject'] == '' )
1261. {
1262. $this->rdf_parser['top']['subject']=$this->rdf_get_base();
1263. }

1265. if( $bag_id )
1266. {
1267. $this->_resolve_id( $bag_id, $id_buffer );
1268. $this->rdf_parser['top']['bag_id']=$id_buffer;
1269. }

1271. // only report the type for non-rdf:Description elements.
1272. if( ($local_name != RDF_DESCRIPTION )
1273. || ( $namespace_uri != RDF_NAMESPACE_URI ) )
1274. {
1275. $type=$namespace_uri;
1276. $type.=$local_name;

1278. $this->_report_statement(
1279. $this->rdf_parser['top']['subject_type'],
1280. $this->rdf_parser['top']['subject'],
1281. RDF_NAMESPACE_URI.RDF_TYPE,
1282. 0,
1283. RDF_OBJECT_TYPE_RESOURCE,
1284. $type,
1285. '',
1286. $this->rdf_parser['top']['bag_id'],
1287. $this->rdf_parser['top']['statements'],
1288. '',
1289. $datatype);

1291. }

1293. // if this element is the child of some property,
1294. //   report the appropriate statement.
1295. if( $parent )
1296. {
1297. if ($this->rdf_parser['top']['subject_type'] == RDF_SUBJECT_TYPE_BNODE)
1298. $objtype = RDF_OBJECT_TYPE_BNODE;
1299. else
1300. $objtype = RDF_OBJECT_TYPE_RESOURCE;

1302. $this->_report_statement(
1303. $parent['parent']['subject_type'],
1304. $parent['parent']['subject'],
1305. $parent['predicate'],
1306. $parent['ordinal'],
1307. $objtype,
1308. $this->rdf_parser['top']['subject'],
1309. '',
1310. $parent['parent']['bag_id'],
1311. $parent['parent']['statements'],
1312. $parent['statement_id'],
1313. $parent['datatype']);

1315. }

1317. if( $this->rdf_parser['top']['has_property_attributes'] )
1318. {
1319. $this->_handle_property_attributes(
1320. $this->rdf_parser['top']['subject_type'],
1321. $this->rdf_parser['top']['subject'],
1322. $attributes,
1323. $this->rdf_parser['top']['xml_lang'],
1324. $this->rdf_parser['top']['bag_id'],
1325. $this->rdf_parser['top']['statements'] );
1326. }
1327. }

1329. /**
1330. * @param string &$namespace_uri
1331. * @param string &$local_name
1332. * @param string &$attributes
1333. * @access    private
1334. */
1335. function _handle_property_element( &$namespace_uri, &$local_name, &$attributes )
1336. {
1337. $buffer='';

1339. $i=0;

1341. $aux=$attributes;
1342. $aux2=Array();
1343. foreach($attributes as $atkey=>$atvalue) {
1344. $aux2[]=$atkey;
1345. $aux2[]=$atvalue;
1346. }
1347. $attributes=$aux2;

1349. $attribute_namespace_uri='';
1350. $attribute_local_name='';
1351. $attribute_value = '';

1353. $resource = NULL;
1354. $statement_id = '';
1355. $bag_id = '';
1356. $parse_type = '';
1357. $node_id = '';
1358. $datatype = '';

1360. $this->rdf_parser['top']['ordinal'] = 0;

1362. if( $namespace_uri == RDF_NAMESPACE_URI )
1363. {
1364. if( ! $this->_is_rdf_property_element( $local_name ) )
1365. {
1366. $msg = 'unknown or out of context rdf property element: '.$local_name;

1368. if ($this->_is_forbidden_rdf_property_element($local_name))
1369. $this->_report_error($msg);
1370. else
1371. $this->_report_warning($msg);
1372. }

1374. }

1376. $buffer=$namespace_uri;

1378. if( ( $namespace_uri == RDF_NAMESPACE_URI )
1379. &&  ( $local_name == RDF_LI ) )
1380. {
1381. $this->rdf_parser['top']['parent']['members']++;
1382. $this->rdf_parser['top']['ordinal'] = $this->rdf_parser['top']['parent']['members'];

1384. $this->rdf_parser['top']['ordinal']=$this->rdf_parser['top']['ordinal'];

1386. $buffer.='_'.$this->rdf_parser['top']['ordinal'];

1388. }
1389. else
1390. {
1391. $buffer.=$local_name;
1392. }

1394. $this->rdf_parser['top']['predicate']=$buffer;

1396. $this->rdf_parser['top']['has_property_attributes'] = false;
1397. $this->rdf_parser['top']['has_member_attributes'] = false;


1400. for( $i = 0; isset($attributes[$i]); $i += 2 )
1401. {
1402. $this->_split_name(
1403. $attributes[$i],
1404. $buffer,
1405. $attribute_namespace_uri,
1406. $attribute_local_name );

1408. $attribute_value = $attributes[$i + 1];

1410. // if the attribute is not in any namespace
1411. //   or the attribute is in the RDF namespace
1412. if( ( $attribute_namespace_uri == '' )
1413. || (  $attribute_namespace_uri == RDF_NAMESPACE_URI ) )
1414. {
1415. if( ( $attribute_local_name == RDF_ID )  )
1416. {
1417. $statement_id = $attribute_value;
1418. }
1419. else if( $attribute_local_name == RDF_PARSE_TYPE )
1420. {
1421. $parse_type = $attribute_value;
1422. }
1423. else if(  $attribute_local_name == RDF_RESOURCE )
1424. {
1425. $resource = $attribute_value;
1426. }
1427. else if(  $attribute_local_name == RDF_NODEID )
1428. {
1429. $node_id = $attribute_value;
1430. }
1431. else if(  $attribute_local_name == RDF_BAG_ID )
1432. {
1433. $bag_id = $attribute_value;
1434. }
1435. else if(  $attribute_local_name == RDF_DATATYPE )
1436. {
1437. $datatype = $attribute_value;
1438. $this->rdf_parser['top']['datatype'] = $attribute_value;
1439. }
1440. else if( $this->_is_rdf_property_attribute( $attribute_local_name ) )
1441. {
1442. $this->rdf_parser['top']['has_property_attributes'] = true;
1443. }
1444. else
1445. {
1446. $this->_report_warning('unknown rdf attribute: '.$attribute_local_name );
1447. return;
1448. }
1449. }
1450. else if(  $attribute_namespace_uri == XML_NAMESPACE_URI  )
1451. {
1452. if( $attribute_local_name == XML_LANG  )
1453. {
1454. $this->rdf_parser['top']['xml_lang'] = $attribute_value;
1455. }
1456. elseif ($attribute_local_name == 'base')
1457. {
1458. $this->rdf_parser['top']['element_base_uri'] = $attribute_value;
1459. }
1460. }
1461. else if( $attribute_namespace_uri )
1462. {
1463. $this->rdf_parser['top']['has_property_attributes'] = true;
1464. }
1465. }

1467. if( $statement_id )
1468. {
1469. $this->_resolve_id($statement_id, $buffer );
1470. $this->rdf_parser['top']['statement_id']=$buffer;
1471. }

1473. if ($node_id)
1474. {
1475. $this->is_valid_id($node_id);

1477. if ($resource)
1478. {
1479. $this->_report_error('nodeID and resource are mutually exclusive');
1480. }
1481. if ($statement_id)
1482. {
1483. // reify statement
1484. $this->_report_statement(
1485. $this->rdf_parser['top']['parent']['subject_type'],
1486. $this->rdf_parser['top']['parent']['subject'],
1487. $this->rdf_parser['top']['predicate'],
1488. $this->rdf_parser['top']['ordinal'],
1489. RDF_OBJECT_TYPE_BNODE,
1490. $node_id,
1491. '',
1492. $this->rdf_parser['top']['parent']['bag_id'],
1493. $this->rdf_parser['top']['parent']['statements'],
1494. $this->rdf_parser['top']['statement_id'],
1495. '');
1496. $statement_id = '';
1497. }
1498. else
1499. {
1500. $this->_report_statement(
1501. $this->rdf_parser['top']['parent']['subject_type'],
1502. $this->rdf_parser['top']['parent']['subject'],
1503. $this->rdf_parser['top']['predicate'],
1504. $this->rdf_parser['top']['ordinal'],
1505. RDF_OBJECT_TYPE_BNODE,
1506. $node_id,
1507. '',
1508. $this->rdf_parser['top']['parent']['bag_id'],
1509. $this->rdf_parser['top']['parent']['statements'],
1510. '',
1511. $datatype );
1512. }

1514. $this->rdf_parser['top']['state'] = IN_PROPERTY_EMPTY_RESOURCE;
1515. }

1517. if( $parse_type )
1518. {
1519. if( $resource ) {
1520. $this->_report_error('property elements with rdf:parseType do not allow rdf:resource' );
1521. }

1523. if( $bag_id ) {
1524. $this->_report_warning('property elements with rdf:parseType do not allow rdf:bagID' );
1525. return;
1526. }

1528. if( $this->rdf_parser['top']['has_property_attributes'] )
1529. {
1530. $this->_report_error('property elements with rdf:parseType do not allow property attributes');
1531. return;
1532. }

1534. if(  $attribute_value == RDF_PARSE_TYPE_RESOURCE )
1535. {
1536. $this->_generate_anonymous_uri( $buffer );
1537. // since we are sure that this is now a resource property we can report it
1538. $this->_report_statement(
1539. $this->rdf_parser['top']['parent']['subject_type'],
1540. $this->rdf_parser['top']['parent']['subject'],
1541. $this->rdf_parser['top']['predicate'],
1542. 0,
1543. RDF_OBJECT_TYPE_BNODE,
1544. $buffer,
1545. '',
1546. $this->rdf_parser['top']['parent']['bag_id'],
1547. $this->rdf_parser['top']['parent']['statements'],
1548. $this->rdf_parser['top']['statement_id'],
1549. $datatype );

1551. $this->_push_element( );

1553. $this->rdf_parser['top']['state'] = IN_PROPERTY_PARSE_TYPE_RESOURCE;
1554. $this->rdf_parser['top']['subject_type'] = RDF_SUBJECT_TYPE_BNODE;
1555. $this->rdf_parser['top']['subject']=$buffer;
1556. $this->rdf_parser['top']['bag_id']='';
1557. $this->rdf_parser['top']['datatype']= $datatype;

1559. }
1560. elseif (  $attribute_value == RDF_PARSE_TYPE_LITERAL )
1561. {
1562. $this->rdf_parser['top']['state'] = IN_PROPERTY_PARSE_TYPE_LITERAL;
1563. $this->rdf_parser['top']['datatype']= RDF_NAMESPACE_URI .RDF_XMLLITERAL;
1564. $this->rdf_parser['xml_literal']['buffer'] = '';
1565. $this->rdf_parser['xml_literal']['depth'] = 0;
1566. }
1567. elseif ($attribute_value == RDF_PARSE_TYPE_COLLECTION)
1568. {
1569. $this->_generate_anonymous_uri( $buffer );
1570. $this->_report_statement(
1571. $this->rdf_parser['top']['parent']['subject_type'],
1572. $this->rdf_parser['top']['parent']['subject'],
1573. $this->rdf_parser['top']['predicate'],
1574. 0,
1575. RDF_OBJECT_TYPE_BNODE,
1576. $buffer,
1577. '',
1578. $this->rdf_parser['top']['parent']['bag_id'],
1579. $this->rdf_parser['top']['parent']['statements'],
1580. $this->rdf_parser['top']['statement_id'],
1581. $datatype );

1583. $this->rdf_parser['top']['state'] = IN_PROPERTY_PARSE_TYPE_COLLECTION;
1584. $this->rdf_parser['top']['collection']['first_blank_node_id'] = $buffer;
1585. }

1587. else
1588. {

1590. $this->_report_statement(
1591. $this->rdf_parser['top']['parent']['subject_type'],
1592. $this->rdf_parser['top']['parent']['subject'],
1593. $this->rdf_parser['top']['predicate'],
1594. 0,
1595. RDF_OBJECT_TYPE_XML,
1596. '',
1597. '',
1598. $this->rdf_parser['top']['parent']['bag_id'],
1599. $this->rdf_parser['top']['parent']['statements'],
1600. $this->rdf_parser['top']['statement_id'],
1601. $datatype );

1603. $this->rdf_parser['top']['state'] = IN_PROPERTY_PARSE_TYPE_LITERAL;
1604. }
1605. }
1606. else if( $resource !== NULL || $bag_id || $this->rdf_parser['top']['has_property_attributes'] )
1607. {
1608. if( $resource !== NULL )
1609. {
1610. $subject_type = RDF_SUBJECT_TYPE_URI;
1611. $this->_resolve_uri_reference( $this->rdf_get_base(), $resource, $buffer );
1612. $object_type=RDF_OBJECT_TYPE_RESOURCE;
1613. }
1614. else
1615. {
1616. $subject_type = RDF_SUBJECT_TYPE_BNODE;
1617. $this->_generate_anonymous_uri( $buffer );
1618. $object_type=RDF_OBJECT_TYPE_BNODE;
1619. }
1620. $this->rdf_parser['top']['state'] = IN_PROPERTY_EMPTY_RESOURCE;

1622. // since we are sure that this is now a resource property we can report it.
1623. $this->_report_statement(
1624. $this->rdf_parser['top']['parent']['subject_type'],
1625. $this->rdf_parser['top']['parent']['subject'],
1626. $this->rdf_parser['top']['predicate'],
1627. $this->rdf_parser['top']['ordinal'],
1628. $object_type,
1629. $buffer,
1630. '',
1631. $this->rdf_parser['top']['parent']['bag_id'],
1632. $this->rdf_parser['top']['parent']['statements'],
1633. $this->rdf_parser['top']['statement_id'],
1634. $datatype ); // should we allow IDs?

1636. if( $bag_id )
1637. {
1638. $this->_resolve_id( $bag_id, $buffer );
1639. $this->rdf_parser['top']['bag_id']=$buffer;
1640. }

1642. if( $this->rdf_parser['top']['has_property_attributes'] )
1643. {
1644. $this->_handle_property_attributes(
1645. $subject_type,
1646. $buffer,
1647. $attributes,
1648. $this->rdf_parser['top']['xml_lang'],
1649. $this->rdf_parser['top']['bag_id'],
1650. $this->rdf_parser['top']['statements'] );
1651. }
1652. }
1653. }

1655. /**
1656. * @param string &$namespace_uri
1657. * @param string &$local_name
1658. * @param string &$attributes
1659. * @access    private
1660. */
1661. function _handle_collection_element(&$namespace_uri, &$local_name, &$attributes)
1662. {
1663. $aux2=Array();
1664. foreach($attributes as $atkey=>$atvalue) {
1665. $aux2[]=$atkey;
1666. $aux2[]=$atvalue;
1667. }
1668. $attributes=$aux2;
1669. /* collection construction site
1670. // old:
1671. if (   ($namespace_uri == RDF_NAMESPACE_URI || $namespace_uri == '')
1672. && ($local_name == RDF_DESCRIPTION || $local_name == RDF_LI) )
1673. {
1674. for( $i = 0; isset($attributes[$i]); $i += 2 )
1675. {
1676. $this->_split_name(
1677. $attributes[ $i ],
1678. $attribute,
1679. $attribute_namespace_uri,
1680. $attribute_local_name );

1682. $attribute_value = $attributes[ $i + 1 ];

1684. if( $attribute_namespace_uri == '' || $attribute_namespace_uri == RDF_NAMESPACE_URI  )
1685. {
1686. if( $attribute_local_name == RDF_ABOUT ||
1687. $attribute_local_name == RDF_RESOURCE)
1688. {
1689. $this->rdf_parser['top']['parent']['collection']['object_type'][] = RDF_OBJECT_TYPE_RESOURCE;
1690. }
1691. elseif ( $attribute_local_name == RDF_NODEID ) {
1692. $this->rdf_parser['top']['parent']['collection']['object_type'][] = RDF_OBJECT_TYPE_BNODE;
1693. }
1694. $this->rdf_parser['top']['parent']['collection']['object_label'][] = $attribute_value;
1695. }
1696. }
1697. }
1698. */
1699. // new

1701. for( $i = 0; isset($attributes[$i]); $i += 2 )
1702. {
1703. $this->_split_name(
1704. $attributes[ $i ],
1705. $attribute,
1706. $attribute_namespace_uri,
1707. $attribute_local_name );

1709. $attribute_value = $attributes[ $i + 1 ];

1711. if( $attribute_namespace_uri == '' || $attribute_namespace_uri == RDF_NAMESPACE_URI  )
1712. {
1713. $tmp_subject_type = RDF_SUBJECT_TYPE_URI;
1714. if( $attribute_local_name == RDF_ABOUT ||
1715. $attribute_local_name == RDF_RESOURCE)
1716. {
1717. $this->rdf_parser['top']['parent']['collection']['object_type'][] = RDF_OBJECT_TYPE_RESOURCE;
1718. }
1719. elseif ( $attribute_local_name == RDF_NODEID ) {
1720. $this->rdf_parser['top']['parent']['collection']['object_type'][] = RDF_OBJECT_TYPE_BNODE;
1721. $tmp_subject_type = RDF_SUBJECT_TYPE_BNODE;
1722. }
1723. $id_buffer = '';
1724. $this->_resolve_uri_reference( $this->rdf_get_base(), $attribute_value, $id_buffer );
1725. $this->rdf_parser['top']['parent']['collection']['object_label'][] = $id_buffer;

1727. if (!(   ($namespace_uri == RDF_NAMESPACE_URI || $namespace_uri == '')
1728. && ($local_name == RDF_DESCRIPTION || $local_name == RDF_LI) ))
1729. {
1730. $this->_report_statement(
1731. $tmp_subject_type,
1732. $id_buffer,
1733. RDF_NAMESPACE_URI.RDF_TYPE,
1734. '',
1735. RDF_OBJECT_TYPE_RESOURCE,
1736. $namespace_uri.$local_name,
1737. '',
1738. '',
1739. '',
1740. '',
1741. '');
1742. }
1743. }
1744. }


1747. // collection construction site
1748. }

1750. /**
1751. * @param string &$namespace_uri
1752. * @param string &$local_name
1753. * @param string &$attributes
1754. * @access    private
1755. */
1756. function _handle_xml_start_element(&$namespace_uri, &$local_name, &$attributes)
1757. {
1758. $aux2=Array();
1759. foreach($attributes as $atkey=>$atvalue) {
1760. $aux2[]=$atkey;
1761. $aux2[]=$atvalue;
1762. }
1763. $attributes=$aux2;

1765. $element = '<' .$this->_join_name_and_declare_prefix($namespace_uri, $local_name);

1767. for( $i = 0; isset($attributes[$i]); $i += 2 )
1768. {
1769. $this->_split_name(
1770. $attributes[ $i ],
1771. $attribute,
1772. $attribute_namespace_uri,
1773. $attribute_local_name );

1775. $attribute_value = $attributes[ $i + 1 ];

1777. $element .= ' ' .$this->_join_name_and_declare_prefix($attribute_namespace_uri, $attribute_local_name);
1778. $element .= '=\"' .$attribute_value .'\"';
1779. }
1780. $element .= '>';

1782. $this->rdf_parser['xml_literal']['buffer'] .= $element;
1783. }

1785. /**
1786. * @param string $name
1787. * @access    private
1788. */
1789. function _handle_xml_end_element($name)
1790. {
1791. $buffer='';
1792. $namespace_uri='';
1793. $local_name='';

1795. $this->_split_name(
1796. $name,
1797. $buffer,
1798. $namespace_uri,
1799. $local_name );

1801. $element = '</';

1803. if ($namespace_uri && isset($this->rdf_parser['default_namespace'])
1804. &&$namespace_uri != $this->rdf_parser['default_namespace'])
1805. {
1806. $element .= $this->rdf_parser['namespaces'][$namespace_uri] .':';
1807. }

1809. $element .= $local_name .'>';

1811. $this->rdf_parser['xml_literal']['buffer'] .= $element;
1812. $depth = $this->rdf_parser['xml_literal']['depth']--;

1814. if (isset($this->rdf_parser['xml_literal']['declared_ns']))
1815. foreach ($this->rdf_parser['xml_literal']['declared_ns'] as $prefix => $_depth)
1816. {
1817. if ($depth == $_depth)
1818. unset($this->rdf_parser['xml_literal']['declared_ns'][$prefix]);
1819. }
1820. }

1822. /**
1823. * @param string $namespace_uri
1824. * @param string $local_name
1825. * @access    private
1826. */
1827. function _join_name_and_declare_prefix($namespace_uri, $local_name) {

1829. $name = '';

1831. if ($namespace_uri)
1832. {
1833. if (isset($this->rdf_parser['default_namespace'])
1834. && $namespace_uri == $this->rdf_parser['default_namespace'])
1835. {
1836. $name .= $local_name;

1838. if (!isset($this->rdf_parser['xml_literal']['declared_ns']['_DEFAULT_'])
1839. && $namespace_uri != XML_NAMESPACE_URI)
1840. {
1841. $name .= ' xmlns=' . '\"' .$namespace_uri .'\"';

1843. $this->rdf_parser['xml_literal']['declared_ns']['_DEFAULT_']
1844. = $this->rdf_parser['xml_literal']['depth'];
1845. }
1846. }
1847. else
1848. {
1849. $ns_prefix = $this->rdf_parser['namespaces'][$namespace_uri];
1850. $name .= $ns_prefix .':' .$local_name;

1852. if (!isset($this->rdf_parser['xml_literal']['declared_ns'][$ns_prefix])
1853. && $namespace_uri != XML_NAMESPACE_URI)
1854. {
1855. $name .= " xmlns:$ns_prefix=" . '\"' .$namespace_uri .'\"';

1857. $this->rdf_parser['xml_literal']['declared_ns'][$ns_prefix]
1858. = $this->rdf_parser['xml_literal']['depth'];
1859. }
1860. }

1862. }
1863. else
1864. $name .= $local_name;

1866. return $name;

1868. }

1870. /**
1871. * @access    private
1872. */
1873. function _end_collection() {

1875. if (isset($this->rdf_parser['top']['collection']))
1876. {

1878. $subject = $this->rdf_parser['top']['collection']['first_blank_node_id'];

1880. for ($i=0; isset($this->rdf_parser['top']['collection']['object_label'][$i]); $i++)
1881. {

1883. $this->_report_statement(
1884. RDF_SUBJECT_TYPE_BNODE,
1885. $subject,
1886. RDF_NAMESPACE_URI.RDF_FIRST,
1887. '',
1888. $this->rdf_parser['top']['collection']['object_type'][$i],
1889. $this->rdf_parser['top']['collection']['object_label'][$i],
1890. '',
1891. '',
1892. '',
1893. '',
1894. '');

1896. if (!isset($this->rdf_parser['top']['collection']['object_label'][$i+1]))
1897. {
1898. $obj_type_2 = RDF_OBJECT_TYPE_RESOURCE;
1899. $object_2 = RDF_NAMESPACE_URI.RDF_NIL;
1900. }
1901. else
1902. {
1903. $obj_type_2= RDF_OBJECT_TYPE_BNODE;
1904. $this->_generate_anonymous_uri($object_2);
1905. }


1908. $this->_report_statement(
1909. RDF_SUBJECT_TYPE_BNODE,
1910. $subject,
1911. RDF_NAMESPACE_URI.RDF_REST,
1912. '',
1913. $obj_type_2,
1914. $object_2,
1915. '',
1916. '',
1917. '',
1918. '',
1919. '');

1921. $subject = $object_2;
1922. }
1923. }
1924. }

1926. /**
1927. * @param string $parser
1928. * @param string $name
1929. * @param string $attributes
1930. * @access    private
1931. */
1932. function _start_element_handler($parser, $name, $attributes )
1933. {
1934. $buffer='';

1936. $namespace_uri='';
1937. $local_name='';

1939. $this->_push_element();


1942. $this->_split_name(
1943. $name,
1944. $buffer,
1945. $namespace_uri,
1946. $local_name );

1948. switch( $this->rdf_parser['top']['state'] )
1949. {
1950. case IN_TOP_LEVEL:
1951. // set base_uri, if possible
1952. foreach ($attributes as $key => $value) {
1953. if($key == XML_NAMESPACE_URI . NAMESPACE_SEPARATOR_CHAR . 'base') {
1954. $this->rdf_parser['base_uri'] = $value;
1955. $this->rdf_parser['document_base_uri'] = $value;

1957. $c = substr($value, strlen($value)-1 ,1);
1958. if (!($c=='#' || $c==':' || $c=='/' || $c=="\\"))
1959. $this->rdf_parser['normalized_base_uri'] = $value . '#';
1960. else
1961. $this->rdf_parser['normalized_base_uri'] = $value;

1963. }
1964. elseif ($key == XML_NAMESPACE_URI . NAMESPACE_SEPARATOR_CHAR .'lang')
1965. $this->rdf_parser['document_xml_lang'] = $value;
1966. echo "";
1967. }


1970. if( RDF_NAMESPACE_URI.NAMESPACE_SEPARATOR_STRING.RDF_RDF == $name )
1971. {
1972. $this->rdf_parser['top']['state'] = IN_RDF;

1974. break;
1975. }
1976. case IN_RDF:
1977. $this->rdf_parser['top']['state'] = IN_DESCRIPTION;
1978. $this->_handle_resource_element( $namespace_uri, $local_name, $attributes, '' );
1979. break;
1980. case IN_DESCRIPTION:
1981. case IN_PROPERTY_PARSE_TYPE_RESOURCE:
1982. $this->rdf_parser['top']['state'] = IN_PROPERTY_UNKNOWN_OBJECT;
1983. $this->_handle_property_element( $namespace_uri, $local_name, $attributes );
1984. break;
1985. case IN_PROPERTY_PARSE_TYPE_COLLECTION:
1986. $this->_handle_collection_element($namespace_uri, $local_name, $attributes);
1987. break;
1988. case IN_PROPERTY_UNKNOWN_OBJECT:
1989. /* if we're in a property with an unknown object type and we encounter
1990. an element, the object must be a resource, */
1991. $this->rdf_parser['top']['data']='';
1992. $this->rdf_parser['top']['parent']['state'] = IN_PROPERTY_RESOURCE;
1993. $this->rdf_parser['top']['state'] = IN_DESCRIPTION;
1994. $this->_handle_resource_element(
1995. $namespace_uri,
1996. $local_name,
1997. $attributes,
1998. $this->rdf_parser['top']['parent'] );
1999. break;
2000. case IN_PROPERTY_LITERAL:
2001. $this->_report_warning( 'no markup allowed in literals' );
2002. break;
2003. case IN_PROPERTY_PARSE_TYPE_LITERAL:
2004. $this->rdf_parser['top']['state'] = IN_XML;
2005. /* fall through */
2006. case IN_XML:
2007. $this->rdf_parser['xml_literal']['depth']++;
2008. $this->_handle_xml_start_element($namespace_uri, $local_name, $attributes);
2009. break;
2010. case IN_PROPERTY_RESOURCE:
2011. $this->_report_warning(
2012. 'only one element allowed inside a property element' );
2013. break;
2014. case IN_PROPERTY_EMPTY_RESOURCE:
2015. $this->_report_warning(
2016. 'no content allowed in property with rdf:resource, rdf:bagID, or property attributes' );
2017. break;
2018. case IN_UNKNOWN:
2019. break;
2020. }
2021. }

2023. /**
2024. property elements with text only as content set the state to
2025. IN_PROPERTY_LITERAL. as character data is received from expat,
2026. it is saved in a buffer and reported when the end tag is
2027. received.
2028. * @access    private
2029. */
2030. function _end_literal_property()
2031. {
2032. if(!isset($this->rdf_parser['top']['statement_id'])) {
2033. $this->rdf_parser['top']['statement_id']='';
2034. }
2035. if(!isset($this->rdf_parser['top']['parent']['subject_type'])) {
2036. $this->rdf_parser['top']['parent']['subject_type']='';
2037. }
2038. if(!isset($this->rdf_parser['top']['parent']['subject'])) {
2039. $this->rdf_parser['top']['parent']['subject']='';
2040. }
2041. if(!isset($this->rdf_parser['top']['parent']['bag_id'])) {
2042. $this->rdf_parser['top']['parent']['bag_id']='';
2043. }
2044. if(!isset($this->rdf_parser['top']['parent']['statements'])) {
2045. $this->rdf_parser['top']['parent']['statements']=0;
2046. }
2047. if(!isset($this->rdf_parser['top']['predicate'])) {
2048. $this->rdf_parser['top']['predicate']='';
2049. }
2050. if(!isset($this->rdf_parser['top']['datatype'])) {
2051. $this->rdf_parser['top']['datatype']='';
2052. }
2053. if(!isset($this->rdf_parser['top']['ordinal'])) {
2054. $this->rdf_parser['top']['ordinal']=0;
2055. }
2056. $this->_report_statement(
2057. $this->rdf_parser['top']['parent']['subject_type'],
2058. $this->rdf_parser['top']['parent']['subject'],
2059. $this->rdf_parser['top']['predicate'],
2060. $this->rdf_parser['top']['ordinal'],
2061. RDF_OBJECT_TYPE_LITERAL,
2062. $this->rdf_parser['top']['data'],
2063. $this->rdf_parser['top']['xml_lang'],
2064. $this->rdf_parser['top']['parent']['bag_id'],
2065. $this->rdf_parser['top']['parent']['statements'],
2066. $this->rdf_parser['top']['statement_id'],
2067. $this->rdf_parser['top']['datatype']);

2069. }

2071. /**
2072. * @param string $parser
2073. * @param string $name
2074. * @access    private
2075. */
2076. function _end_element_handler( $parser, $name )
2077. {
2078. switch( $this->rdf_parser['top']['state'] )
2079. {
2080. case IN_TOP_LEVEL:
2081. break;
2082. case IN_XML:
2083. $this->_handle_xml_end_element($name);
2084. break;
2085. case IN_PROPERTY_UNKNOWN_OBJECT:
2086. case IN_PROPERTY_LITERAL:
2087. $this->_end_literal_property( );
2088. break;
2089. case IN_PROPERTY_PARSE_TYPE_RESOURCE:
2090. $this->_pop_element(  );
2091. break;
2092. case IN_PROPERTY_PARSE_TYPE_LITERAL:
2093. //        $search =  array((0) => chr(10), (1) => chr(13), (2) => chr(9));
2094. //        $replace = array((0) => '\n'   , (1) => '\r'   , (2) => '\t');
2095. //        $this->rdf_parser["xml_literal"]["buffer"]
2096. //            = str_replace($search, $replace, $this->rdf_parser["xml_literal"]["buffer"]);

2098. $this->rdf_parser['top']['data'] = $this->rdf_parser['xml_literal']['buffer'];
2099. $this->_end_literal_property();
2100. $this->rdf_parser['xml_literal']['buffer'] = '';

2102. break;
2103. case IN_PROPERTY_PARSE_TYPE_COLLECTION:
2104. $this->_end_collection();
2105. break;
2106. case IN_RDF:
2107. case IN_DESCRIPTION:
2108. case IN_PROPERTY_RESOURCE:
2109. case IN_PROPERTY_EMPTY_RESOURCE:
2110. case IN_UNKNOWN:
2111. break;
2112. }

2114. $this->_pop_element();
2115. }

2117. /**
2118. * @param string $parser
2119. * @param string $s
2120. * @access    private
2121. */
2122. function _character_data_handler( $parser,$s)
2123. {
2124. $len=strlen($s);
2125. switch( $this->rdf_parser['top']['state'] )
2126. {
2127. case IN_PROPERTY_LITERAL:
2128. case IN_PROPERTY_UNKNOWN_OBJECT:
2129. if( isset($this->rdf_parser['top']['data']) )
2130. {
2131. $n = strlen( $this->rdf_parser['top']['data'] );
2132. $this->rdf_parser['top']['data'].= $s;

2134. }
2135. else
2136. {
2137. $this->rdf_parser['top']['data']=$s;
2138. }

2140. if( $this->rdf_parser['top']['state'] == IN_PROPERTY_UNKNOWN_OBJECT )
2141. {
2142. /* look for non-whitespace */
2143. for( $i = 0; (( $i < $len ) && (  ereg(" |\n|\t",$s{ $i }) )); $i++ );
2144. /* if we found non-whitespace, this is a literal */
2145. if( $i < $len )
2146. {
2147. $this->rdf_parser['top']['state'] = IN_PROPERTY_LITERAL;
2148. }
2149. }

2151. break;
2152. case IN_TOP_LEVEL:
2153. break;
2154. case IN_PROPERTY_PARSE_TYPE_LITERAL:
2155. case IN_XML:
2156. $this->rdf_parser['xml_literal']['buffer'] .= $s;
2157. break;
2158. case IN_RDF:
2159. case IN_DESCRIPTION:
2160. case IN_PROPERTY_RESOURCE:
2161. case IN_PROPERTY_EMPTY_RESOURCE:
2162. case IN_PROPERTY_PARSE_TYPE_RESOURCE:
2163. case IN_UNKNOWN:
2164. break;
2165. }
2166. }


2169. /**
2170. * Adds a new statement to the model
2171. * This method is called by generateModel().
2172. *
2173. * @access    private
2174. * @param    string    &$user_data
2175. * @param    string    $subject_type
2176. * @param    string    $subject
2177. * @param    string    $predicate
2178. * @param    string    $ordinal
2179. * @param    string    $object_type
2180. * @param    string    $object
2181. * @param    string    $xml_lang )
2182. * @return    object MemModel
2183. */
2184. function add_statement_to_model(
2185. &$user_data,
2186. $subject_type,
2187. $subject,
2188. $predicate,
2189. $ordinal,
2190. $object_type,
2191. $object,
2192. $xml_lang,
2193. $datatype )
2194. {

2196. // ParseUnicode
2197. if(UNIC_RDF){
2198. $subject=$this->str2unicode_nfc($subject);
2199. $predicate=$this->str2unicode_nfc($predicate);
2200. $object=$this->str2unicode_nfc($object);
2201. }

2203. //create subject
2204. if ($subject_type == RDF_SUBJECT_TYPE_BNODE)
2205. $objsub = new BlankNode($subject);
2206. else
2207. $objsub = new Resource($subject);

2209. // create predicate
2210. $objpred = new Resource($predicate);

2212. // create object
2213. if (($object_type == RDF_OBJECT_TYPE_RESOURCE) || ($object_type == RDF_OBJECT_TYPE_BNODE)) {
2214. if ($object_type == RDF_OBJECT_TYPE_BNODE)
2215. $objobj = new BlankNode($object);
2216. else
2217. $objobj = new Resource($object);
2218. } else {

2220. $objobj = new Literal($object);
2221. if ($datatype != '') {
2222. $objobj->setDatatype($datatype);
2223. }
2224. elseif ($xml_lang !='') {
2225. $objobj->setLanguage($xml_lang);
2226. }
2227. }

2229. // create statement
2230. $statement = new Statement($objsub, $objpred, $objobj);

2232. // add statement to model
2233. if(CREATE_MODEL_WITHOUT_DUPLICATES == TRUE){
2234. $this->model->addWithoutDuplicates($statement);
2235. }else
2236. $this->model->add($statement);
2237. }


2240. /* public functions */

2242. /**
2243. * Generates a new MemModel from a URI, a file or from memory.
2244. * If you want to parse an RDF document, pass the URI or location in the filesystem
2245. * of the RDF document. You can also pass RDF code direct to the function. If you pass
2246. * RDF code directly to the parser and there is no xml:base included, you should set
2247. * the base URI manually using the optional second parameter $rdfBaseURI.
2248. * Make sure that here are proper namespace declarations in your input document.
2249. *
2250. * @access    public
2251. * @param        string     $base
2252. * @param     boolean  $rdfBaseURI
2253. * @return    object MemModel
2254. */
2255. function & generateModel($base,$rdfBaseURI = false, $model = false) {

2257. // Check if $base is a URI or filename or a string containing RDF code.
2258. if (substr(ltrim($base),0 ,1) != '<') {

2260. // $base is URL or filename
2261. $this->model = $model?$model:new MemModel($base);

2263. $input = fopen($base,'r') or die("RDF Parser: Could not open File: $base. Stopped parsing.");
2264. $this->rdf_parser_create( NULL );
2265. $this->rdf_set_base($base);
2266. $done=false;
2267. while(!$done)
2268. {
2269. $buf = fread( $input, 512 );
2270. $done = feof($input);

2272. if ( ! $this->rdf_parse( $buf, feof($input) ) )
2273. {
2274. $err_code = xml_get_error_code( $this->rdf_get_xml_parser());
2275. $line = xml_get_current_line_number($this->rdf_get_xml_parser() );
2276. $errmsg = RDFAPI_ERROR . '(class: parser; method: generateModel): XML-parser-error ' . $err_code .' in Line ' . $line .' of input document.';
2277. trigger_error($errmsg, E_USER_ERROR);
2278. }
2279. }
2280. /* close file. */
2281. fclose( $input );

2283. } else {
2284. // $base is RDF string
2285. $this->model = $model?$model:new MemModel($base);

2287. $this->rdf_parser_create( NULL );

2289. if ($rdfBaseURI!==false)
2290. {
2291. $this->rdf_set_base($rdfBaseURI);
2292. } else
2293. {
2294. $this->rdf_set_base( NULL );
2295. }

2297. if ( ! $this->rdf_parse( $base, TRUE ) )
2298. {
2299. $err_code = xml_get_error_code( $this->rdf_get_xml_parser());
2300. $line = xml_get_current_line_number($this->rdf_get_xml_parser() );
2301. $errmsg = RDFAPI_ERROR . '(class: parser; method: generateModel): XML-parser-error ' . $err_code .' in Line ' . $line .' of input document.';
2302. trigger_error($errmsg, E_USER_ERROR);
2303. }
2304. }
2305. // base_uri could have changed while parsing
2306. $this->model->setBaseURI($this->rdf_parser['base_uri']);

2308. if(isset($this->rdf_parser['namespaces'])){
2309. $this->model->addParsedNamespaces($this->rdf_parser['namespaces']);
2310. }

2312. $this->rdf_parser_free();

2314. return $this->model;

2316. }

2318. /**
2319. * @param        string     $encoding
2320. * @access    private
2321. */
2322. function rdf_parser_create( $encoding )
2323. {

2325. $parser = xml_parser_create_ns( $encoding, NAMESPACE_SEPARATOR_CHAR );

2327. xml_parser_set_option($parser,XML_OPTION_CASE_FOLDING,0);
2328. $this->rdf_parser['xml_parser'] = $parser;

2330. xml_set_object($this->rdf_parser['xml_parser'], $this);
2331. xml_set_element_handler( $this->rdf_parser['xml_parser'], '_start_element_handler', '_end_element_handler' );
2332. xml_set_character_data_handler( $this->rdf_parser['xml_parser'], '_character_data_handler' );
2333. xml_set_start_namespace_decl_handler($this->rdf_parser['xml_parser'], '_start_ns_declaration_handler');

2335. return $this->rdf_parser;
2336. }

2338. /**
2339. * @param    resource    &$parser
2340. * @param   string        $ns_prefix
2341. * @param    string        $ns_uri
2342. * @access    private
2343. */
2344. function _start_ns_declaration_handler(&$parser, $ns_prefix, $ns_uri)
2345. {
2346. if (!$ns_prefix)
2347. $this->rdf_parser['default_namespace'] = $ns_uri;
2348. else
2349. $this->rdf_parser['namespaces'][$ns_uri] = $ns_prefix;
2350. }


2353. /**
2354. * @access    private
2355. */
2356. function rdf_parser_free( )
2357. {
2358. $z=3;

2360. $this->rdf_parser['base_uri']='';
2361. $this->rdf_parser['document_base_uri'] = '';

2363. unset( $this->rdf_parser );
2364. }

2366. /**
2367. * @param        string     $s
2368. * @param        string     $is_final
2369. * @access    private
2370. */
2371. function rdf_parse( $s, $is_final )
2372. {
2373. return XML_Parse( $this->rdf_parser['xml_parser'], $s, $is_final );
2374. }

2376. /**
2377. * @access    private
2378. */
2379. function rdf_get_xml_parser()
2380. {
2381. return ( $this->rdf_parser['xml_parser']);
2382. }

2384. /**
2385. * @param        string     $base
2386. * @access    private
2387. */
2388. function rdf_set_base($base )
2389. {

2391. $this->rdf_parser['base_uri']=$base;

2393. $c = substr($base, strlen($base)-1 ,1);
2394. if (!($c=='#' || $c==':' || $c=='/' || $c=="\\"))
2395. $this->rdf_parser['normalized_base_uri'] = $base . '#';
2396. else
2397. $this->rdf_parser['normalized_base_uri'] = $base;

2399. return 0;
2400. }

2402. /**
2403. * @access    private
2404. */
2405. function rdf_get_base()
2406. {
2407. if ($this->rdf_parser['top']['element_base_uri'])
2408. return $this->rdf_parser['top']['element_base_uri'];
2409. else
2410. return $this->rdf_parser['base_uri'];
2411. }


2414. } // end: rdf_parser

2416. ?>
```

Documentation generated on Thu, 7 Jul 2005 13:42:56 +0200 by phpDocumentor 1.3.0RC3
